# Health Emergency and Disaster Risk Management Workforce Development Strategies: Delphi Consensus Study

**DOI:** 10.1017/S1049023X22001467

**Published:** 2022-12

**Authors:** Kevin K.C. Hung, Makiko K. MacDermot, Emily Y.Y. Chan, Sonoe Mashino, Satchit Balsari, Gregory R. Ciottone, Francesco Della Corte, Marcelo F. Dell’Aringa, Shinichi Egawa, Bettina D. Evio, Alexander Hart, Tadashi Ishii, Luca Ragazzoni, Hiroyuki Sasaki, Joseph Harold Walline, Chi S. Wong, Saurabh Dalal, Ryoma Kayano, Jonathan Abrahams, Qudsia Huda, Colin A. Graham

**Affiliations:** 1. Accident and Emergency Medicine Academic Unit, Chinese University of Hong Kong, Hong Kong, China; 2. Collaborating Centre for Oxford University and Chinese University of Hong Kong for Disaster and Medical Humanitarian Response (CCOUC), School of Public Health and Primary Care, The Chinese University of Hong Kong, Hong Kong, China; 3. Research Institute of Nursing Care for People and Community, University of Hyogo, Akashi, Japan; 4. Beth Israel Deaconess Medical Center, Boston, Massachusetts USA; 5. Department of Global Health and Population, Harvard T.H. Chan School of Public Health, Boston, Massachusetts USA; 6. Harvard Medical School, Boston, Massachusetts USA; 7. CRIMEDIM—Center for Research and Training in Disaster Medicine, Humanitarian Aid and Global Health, Università del Piemonte Orientale, Novara, Italy; 8. Division of International Cooperation for Disaster Medicine, International Research Institute of Disaster Science (IRIDeS), Tohoku University, Miyagi, Japan; 9. College of Nursing, University of the Philippines Manila, Manila, Philippines; 10. University of Connecticut School of Medicine, Farmington, Connecticut USA; 11. Department of Emergency Medicine, Hartford Hospital, Hartford, Connecticut USA; 12. Department of Educational and Support for Regional Medicine, Tohoku University Hospital, Miyagi, Japan; 13. Department of Emergency Medicine, The Pennsylvania State University, College of Medicine, Hershey, Pennsylvania USA; 14. World Health Organization Country Office, New Delhi, India; 15. World Health Organization Centre for Health Development, Kobe, Japan; 16. Disaster Risk Reduction and Resilience Unit, Health Security Preparedness Department, WHO Health Emergencies Program, World Health Organization, Geneva, Switzerland

**Keywords:** developed countries, developing countries, disasters, health workforce, risk management

## Abstract

**Introduction:**

Health workforce development is essential for achieving the goals of an effective health system, as well as establishing national Health Emergency and Disaster Risk Management (Health EDRM).

**Study Objective:**

The objective of this Delphi consensus study was to identify strategic recommendations for strengthening the workforce for Health EDRM in low- and middle-income countries (LMIC) and high-income countries (HIC).

**Methods:**

A total of 31 international experts were asked to rate the level of importance (one being strongly unimportant to seven being strongly important) for 46 statements that contain recommendations for strengthening the workforce for Health EDRM. The experts were divided into a LMIC group and an HIC group. There were three rounds of rating, and statements that did not reach consensus (SD ≥ 1.0) proceeded to the next round for further ranking.

**Results:**

In total, 44 statements from the LMIC group and 34 statements from the HIC group attained consensus and achieved high mean scores for importance (higher than five out of seven). The components of the World Health Organization (WHO) Health EDRM Framework with the highest number of recommendations were “Human Resources” (n = 15), “Planning and Coordination” (n = 7), and “Community Capacities for Health EDRM” (n = 6) in the LMIC group. “Policies, Strategies, and Legislation” (n = 7) and “Human Resources” (n = 7) were the components with the most recommendations for the HIC group.

**Conclusion:**

The expert panel provided a comprehensive list of important and actionable strategic recommendations on workforce development for Health EDRM.

## Introduction

As the coronavirus disease 2019 (COVID-19) pandemic has highlighted, disasters and emergencies from all causes can lead to a dramatic loss of human life and other impacts on people’s health. They often present substantial challenges to public health, health systems, and communities.^1–5^ Rising health risks associated with disasters are due to increasing risk drivers, exposures, and vulnerabilities (such as poverty, risk-exacerbating urbanization, aging societies, and climate change).^6–11^ To protect human health and reduce mortality from disasters, strategic planning and actions for disaster risk management are vital to strengthening local and national capacities and systems within and across all levels of society.^12^

The Sendai Framework for Disaster Risk Reduction 2015-2030 (Sendai Framework) placed human health at the center of disaster risk reduction.^13^ In response, the concept of Health Emergency and Disaster Risk Management (Health EDRM) became increasingly important for applying a risk management approach to health risks associated with emergencies and disasters and ensuring that health risks and consequences are integrated into disaster risk management principles, policies, and practice.^14^ The World Health Organization (WHO; Geneva, Switzerland) Health EDRM Framework was published in 2019 to describe comprehensive actions to reduce hazards, exposures, and vulnerabilities and strengthen capacities for prevention, preparedness, response, and recovery.^15^ It emphasized all-hazard, risk-based, people- and community-centered approaches, and highlighted ten core components comprising around 200 functions for effective Health EDRM. These components and functions reflect the wide-range of inter-related and mutually dependent capacities required for resilience-building in health systems, communities, and countries.

### Health EDRM Workforce Development

The Health EDRM workforce comprises actors across the health system with a wide-range of roles and responsibilities that enable hazards, exposures, and vulnerabilities in communities to be reduced and provide the capacity for preparedness, response, and recovery for emerging and actual events. In order to strengthen this workforce to implement Health EDRM, long-term strategic planning for developing and sustaining the entire health workforce, including a focus on capacities to manage the risks of emergencies and disasters, is essential.^15^ There are several Health EDRM workforce strategies, initiatives, and programs available at the local, national, and global levels.^16,17^

The WHO Emergency Medical Team (EMT) initiative has developed a global registry system for EMTs since 2015 to promote minimum standards for surge capacity.^18,19^ The EMTs must undertake a quality assurance control process to prove that they meet set competencies and standards to provide quality care during emergencies. Global information-sharing initiatives, such as the “OpenWHO,” facilitate online courses and provide practical and evidence-based information on disaster-related topics, such as epidemic preparedness and response.^20^ Workforce management includes planning for personnel surge capacities during an emergency response, training for competency development, as well as occupational health (including the protection, retention, and deployment of staff).^15^

Strong, competent, and well-resourced health workforces working together across different disciplines, sectors, and levels are critical for strengthening Health EDRM programs and building systems that can effectively manage the complex nature of risks in countries and communities. Whether due to varying economic development levels, political will, availability and coverage of health workers, or the dynamic risk environment to personnel, establishing and sustaining such programs can be an immense challenge.

### Health EDRM Workforce Development Research Project

In 2018, the WHO Health EDRM Research Network identified an urgent research need to address knowledge and evidence gaps in Health EDRM workforce capacity development.^21^ An international research project, composed of 15 researchers and practitioners with extensive knowledge in Health EDRM, started in June 2020.^22^ This project aimed to generate policy ideas based on practice evidence and to create important strategic recommendations for facilitating effective workforce development for Health EDRM at local, national, and international levels.

The overall project utilized multiple research methods, including a multilingual literature review, case studies, and an expert (Delphi) consensus study. The literature review identified evidence gaps and collected information on strengthening the Health EDRM workforce.^23^ At the same time, case studies were collected to illustrate best practices in existing Health EDRM workforce development. These combined sources were subsequently analyzed to create preliminary recommendation statements for the next phase of the study.^24–28^

This paper reports the final part of the overall Health EDRM workforce development project: the Delphi consensus study. The focus of this consensus study was to identify strategic recommendations for strengthening the workforce for Health EDRM in low- and middle-income countries (LMIC) and high-income countries (HIC).

## Methods

### Study Design

**Figure 1.**
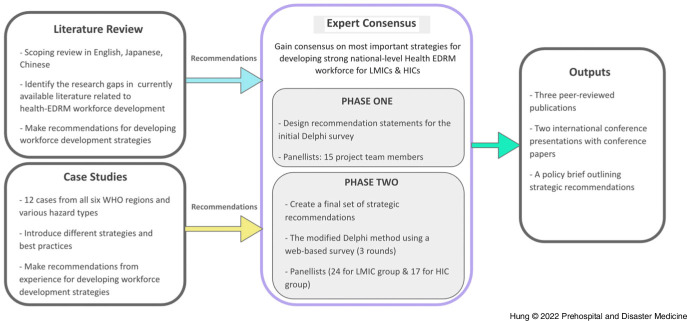
Flow Chart of the Study. Abbreviations: EDRM, emergency and disaster risk management; HIC, high-income country; LMIC, low- and middle-income country; WHO, World Health Organization.

A Delphi-style expert consensus study was conducted. The study was guided by the results from a previous multilingual literature review and multiple case studies (Figure 1). Consensus on the rating of the level of importance for 46 distinct Health EDRM workforce development statements was sought from both LMIC and HIC experts. The Modified Delphi method follows a consultative process to enable a group of diverse experts to make decisions independently and anonymously without a face-to-face meeting.^29–34^ This method is commonly used to aggregate expert opinions for health policy development when available evidence is limited.^30,31^ Three rounds of surveys were conducted at three-week intervals using an iterative web-based survey Stat59 (Build 722fc5, Stat59 Services Ltd; Edmonton, Canada). Ethics approval was obtained from the Chinese University of Hong Kong (Hong Kong, China) Survey and Behavioral Research Ethics Committee (SBRE-21-0152).

### Statement Design (Phase 1)

The study consisted of two phases including statement design (Phase 1) and the Modified Delphi process (Phase 2), as shown in Supplementary Table 1 (available online only). A total of 51 preliminary statements were developed from the literature review and case study analyses. The 51 statements were distributed via a digital survey for review by researcher team members. Fourteen researchers with backgrounds in disaster and emergency medicine, nursing, and public health were asked to keep, delete, or modify each statement by focusing on each statement’s relevance as well as such elements as wording, tone, depth of information, or use of terminology. They could also suggest new statements. All results and comments from the research group were finalized for external expert rating using the Modified Delphi technique (Phase 2).

Thirty statements needed textual adjustment. Five statements were removed due to conceptual overlap with other statements. This resulted in a final list of 46 statements for the Delphi process. These 46 statements were categorized into nine core themes according to the components of the Health EDRM framework:^15^ Policies, Strategies, and Legislation; Planning and Coordination; Human Resources; Information and Knowledge Management; Risk Communications; Health and Related Services; Community Capacities for Health EDRM; and Monitoring and Evaluation. “Health Infrastructure and Logistics,” which includes hospitals and medical supplies, was integrated with “Health and Related Services” for better grouping.

### Modified Delphi Technique (Phase 2)

The same 46 recommendation statements delineated in Phase 1 were used for separate ratings in the LMIC and the HIC groups. Expert panelists (referred to as panelist) were assigned to either the LMIC or the HIC group and each group’s processes were independent of each other. Each panelist was given access to the STAT59 software program to review all statements and were asked to rank the level of importance for each statement on a one-to-seven linear rating scale (one being strongly unimportant to seven being strongly important). In the second and third rounds, each expert was asked to rank each statement again after reviewing the mean group response score for each statement. Panelists were also given an opportunity to suggest a maximum of two new statements in an open textbox at the end of the first and second rounds of the survey.

### Panelist Selection

Phase 2 panelists were selected purposefully based on their professional expertise, prior publication records, and relevant job positions in Health EDRM agencies. The inclusion criteria were: a minimum of five years of experience in a disaster risk management field, or one or more prior publications (eg, a first author or corresponding author publication in a peer-reviewed journal on a relevant topic), or relevant positions held in related institutions (eg, a member of the WHO, national disaster authority, or local government department involved in disaster risk management or health workforce development). A minimum of ten panelists were required for both the LMIC and the HIC group.

### Data Analysis

The mean and standard deviation (SD) of the panelist ratings for each statement were the main measurements in this study. Consensus measurement was also a key component of Delphi analyses and data interpretation. However, there is no universal agreement on determining the level of consensus on the importance of the statement.^33^ Previous studies suggested consensus was reached if the target percentage of agreement was over 51%,^35^ 70%,^33,36–38^ or 80%.^39^ In this study, SD <1.0 (from the mean for the statement) was the cut-off point for the level of agreement. This was because 68.2% of values fall within one standard deviation of the mean score in a normal distribution.^40^

In the first and second surveys, recommendations with a SD <1.0 were regarded as having reached consensus and were excluded in subsequent rounds. Remaining recommendations with a SD ≥1.0 were deemed as requiring further consultation and were carried forward to the next round. During the third (and final) survey, all statements with a SD ≤1.0 were deemed as having reached consensus.

In terms of importance of the statements, the mean score of higher than five (out of seven) was regarded as important. Both the consensus and the mean scores were considered together; for example, high level of consensus on a high mean for the ratings.

Both LMIC and HIC groupings were classified by the gross national income per capita according to the World Bank (Washington, DC USA) classification system.^41^ Experts were allocated to the respective groups based on their current work location. Descriptive statistics including percentages for panelist characteristics, mean (and the 95% confidence interval), and SD for the importance of the statements were used.

## Results

Out of the 72 invited experts, 31 (43.1%) participated in the Delphi consensus survey portion of this study. Consent was obtained from each panelist for participation. Eighteen panelists were allocated to the LMIC group and 13 were allocated to the HIC group. Table 1 illustrates the characteristics of experts in the Phase 2 panel.

**Table 1.** Expert Panelist (Phase 2) Profile

	LMIC (n = 18)	HIC (n = 13)
Male Gender	67% (12)	62% (8)
Country	Bangladesh (1) Brazil (2) Ghana (1) India (1) Indonesia (1) Kenya (1) Malaysia (1) Nepal (2) Philippines (4) South Sudan (1) Sri Lanka (2) Thailand (1)	Australia (2) Israel (1) Japan (5) Portugal (1) Switzerland (1) UK (1) USA (2)
Affiliation		
International Governmental Organization	22% (4)	15% (2)
National Government	33% (6)	8% (1)
Non-Governmental Organization	6% (1)	8% (1)
Academic/Research Institution	39% (7)	69% (9)

Abbreviations: HIC, high-income country; LMIC, low- and middle-income country.

There were 18, 17, and 15 respondents in the LMIC group over the three rounds of surveys and 13, 12, and 12 respondents for the HIC group. Supplementary Table 2 (available online only) shows the results of the three rounds of surveys. Overall, 44 out of the 52 (84.6%) statements attained consensus on the rating of the level of importance in the LMIC group, and 34 out of the 53 (64.2%) statements attained consensus for the HIC group.

Table 2 describes the number of statements which had and had not attained consensus in the various Health EDRM components in the LMIC and the HIC groups. The mean score of the rating of these recommendation statements ranged from 5.9 to 6.8 (out of 7.0) for the LMIC group and 5.1 to 6.2 (out of 7.0) for the HIC group. Scores were higher overall in the LMIC than in the HIC group (mean 6.4 versus 5.7; P <.001).

**Table 2.** Numbers of Initial, Added, Attained, and Not Attained Statements in Each Health EDRM Component

	HIC Group					LMIC Group				
		**Added**					**Added**			
**Component and Functions from Health EDRM Framework**	**Initial**	**2nd**	**3rd**	**Attained**	**Not Attained**	**Initial**	**2nd**	**3rd**	**Attained**	**Not Attained**
Policies, Strategies, and Legislation	5	3	1	7	2	5		1	3	3
Planning and Coordination	7	3		3	7	7	2	1	7	3
Human Resources	14			7	7	14	2		15	1
Financial Resources	3			3		3			3	
Information and Knowledge Management	2			2		2			1	1
Risk Communications	3			3		3			3	
Health and Related Services	4			3	1	4			4	
Community Capacities for Health EDRM	6			4	2	6			6	
Monitoring and Evaluation	2			2		2			2	
**Total**	**46**	**6**	**1**	**34**	**19**	**46**	**4**	**2**	**44**	**8**

Abbreviations: EDRM, emergency and disaster risk management; HIC, high-income country; LMIC, low- and middle-income country.

Table 3 and Table 4 provide the full list of statements that attained consensus for the LMIC and the HIC groups, along with mean scores. Statements that did not attain consensus are listed in Supplementary Table 3 and Supplementary Table 4 (both available online only). The components with the highest number of recommendations were “Human Resources” (n = 15), “Planning and Coordination” (n = 7), and “Community Capacities for Health EDRM” (n = 6) in the LMIC group. “Policies, Strategies, and Legislation” (n = 7) and “Human Resources” (n = 7) were components with the most recommendations in the HIC group.

**Table 3.** Statements Attaining Consensus in the LMIC Group

	LMICs Attained Consensus in 44 (out of the total 52) Statements				
**Round**	**Statement**	**n**	**Mean**	**(95% CI)**	**SD**
	**Policies, Strategies, and Legislation (3)**				
1	HEDRM Component 1 Policies, Strategies, and Legislation: It is important to develop and implement comprehensive health workforce policies and strategic plans for health emergencies based on good understanding of national labor market dynamics through regular capacity and gap assessments.	18	6.7	(5.5-7.9)	0.6
1	HEDRM Component 1 Policies, Strategies, and Legislation: It is important to outline comprehensive workforce structures for health emergencies in the national disaster risk management policy and strategy.	18	6.6	(5.0-8.2)	0.8
1	HEDRM Component 1 Policies, Strategies, and Legislation: It is important to develop national-level health workforce database system to assess and manage the national-level health workforce, which should be regularly updated, easily accessible, and ready for use during health emergencies.	18	6.3	(4.5-8.1)	0.9
	**Planning and Coordination (7)**				
1	HEDRM Component 2 Planning and Coordination: It is important to conduct regular health workforce capacity and gap assessments to understand national labor market dynamics for achieving optimal workforces with respect to quantity, quality, and availability during health emergencies.	18	6.7	(5.5-7.9)	0.6
2	HEDRM Component 2 Planning and Coordination: It is important to ensure that all relevant stakeholders of health emergency and disaster risk management are engaged in developing Health EDRM workforce at national level.	17	6.6	(5.0-8.2)	0.8
3	HEDRM Component 2 Planning and Coordination: It is important to ensure that the private sector health workforce is fully engaged and incorporated in the strategy and as well implementation through the “whole of society” approach.	15	6.3	(4.3-8.3)	1.0
2	HEDRM Component 2 Planning and Coordination: It is important to develop emergency response coordination structures and mechanisms with international humanitarian actors to maximize workforce capacity in response planning, and service delivery including deployment and coordination mechanism in a way to support building back better of affected local health system.	17	6.2	(4.8-7.6)	0.7
3	HEDRM Component 2 Planning and Coordination: It is important to define the roles and contributions of the private sector in the development of the health workforce for disaster risk management.	15	6.1	(4.7-7.5)	0.7
1	HEDRM Component 2 Planning and Coordination: It is important to develop strategies for achieving optimal task-shifting, involving national-level planning, investment, training, quality assurance, and coordination with external supporting agencies.	18	6.0	(4.2-7.8)	0.9
3	HEDRM Component 2 Planning and Coordination: It is important to establish health professional license verification mechanism for foreign health professional volunteers in disaster preparedness phase.	15	5.9	(4.3-7.5)	0.8
	**Human Resources (15)**				
1	HEDRM Component 3 Human Resources: It is important to develop competency-based disaster education courses and training programs for all workforce required during health emergencies, including nurses, paramedics, doctors, hospital managers, and administrative workers.	18	6.7	(5.9-7.5)	0.4
1	HEDRM Component 3 Human Resources: It is important to consider a multidisciplinary approach with professionals from various fields for training national/international Emergency Medical Teams to perform effective operations in emergency settings.	18	6.6	(5.6-7.6)	0.5
2	HEDRM Component 3 Human Resources: It is important to develop education courses and training programs for Health EDRM. These courses and programs require clear aims and learning objectives in alignment with country’s needs and should be based on nationally/internationally recognized organization standards.	17	6.6	(5.4-7.8)	0.6
1	HEDRM Component 3 Human Resources: It is important to work in collaboration with existing national/international professional organizations to develop national/regional/international training programs for Health EDRM.	18	6.6	(5.2-8.0)	0.7
1	HEDRM Component 3 Human Resources: It is important to ensure that the content of national-level education courses and training programs for Health EDRM are based on an all-hazards approach to protect the physical, mental, and social well-being of the affected people. However, the content should also include specific health risks and anticipated impact from high-likelihood events in a country for their frontline workers and their first response organizations.	18	6.6	(5.2-8.0)	0.7
2	HEDRM Component 3 Human Resources: It is important to integrate logistic management knowledge and skills into disaster education programs.	18	6.5	(5.1-7.9)	0.7
2	HEDRM Component 3 Human Resources: It is important to identify core competencies for the key emergency management posts in the health ministry and should ensure their alignment with country’s national disaster risk management plan.	17	6.5	(5.1-7.9)	0.7
2	HEDRM Component 3 Human Resources: It is important to implement national-level disaster training courses with multidisciplinary approach to bring different knowledge and expertise together during health emergencies in order to maximize the use of available resources and systems to save people’s lives.	17	6.5	(5.1-7.9)	0.7
2	HEDRM Component 3 Human Resources: It is important to develop national-level education courses and training programs for Health EDRM in compliance with internationally recognized core competency standards adapted according to national health system practice and priorities.	17	6.5	(4.9-8.1)	0.8
1	HEDRM Component 3 Human Resources: It is important to develop a formal mechanism at national level (eg, needs assessment or skill-gap assessment) to assess training needs regularly. Training institutes should periodically evaluate and update these needs-oriented, competency-based curricula and training activities.	18	6.4	(4.8-8.0)	0.8
1	HEDRM Component 3 Human Resources: It is important to identify adapted definitions of workforce in public health emergencies for their country and clearly define their roles and responsibilities. The required characteristics and scope of those involved may differ depending on country’s contexts according to health systems, legal framework, disaster risk management planning and coordination mechanisms, human resource management, financing, and governance mechanisms.	18	6.4	(4.6-8.2)	0.9
1	HEDRM Component 3 Human Resources: It is important to consider a blended learning approach for national-level education courses and training programs for Health EDRM. The combination of different teaching methods such as traditional instructor-led teaching and technology-enhanced learning, table-top exercises, and computerized and real-world simulations is useful to stimulate different sets of skills.	18	6.3	(4.9-7.7)	0.7
1	HEDRM Component 3 Human Resources: It is important to establish a quality assurance mechanism for health EDRM training programs or education courses, by monitoring and evaluating course achievements and by revising the course contents and modes of delivery according to participants’ needs.	18	6.3	(4.5-8.1)	0.9
1	HEDRM Component 3 Human Resources: It is important to prioritize on providing timely payment of salaries and ensuring occupational safety in order to increase retention and motivation.	18	6.2	(4.4-8.0)	0.9
1	HEDRM Component 3 Human Resources: In order to increase the willingness and remove barriers of workers to respond in disasters, it is important to consider developing a system to provide family care support and other modifiable factors.	18	6.2	(4.4-8.0)	0.9
	**Financial Resources (3)**				
1	HEDRM Component 4 Financial Resources: It is important to allocate sufficient financial resources and invest in the health workforce in order to meet current and future needs during health emergencies for a country.	18	6.7	(5.3-8.1)	0.7
1	HEDRM Component 4 Financial Resources: It is important to allocate sufficient contingency funding for increased workforce costs in all phases of disasters.	18	6.3	(4.5-8.1)	0.9
1	HEDRM Component 4 Financial Resources: It is important to establish mechanisms and platforms to document and share the economic impacts of previous disasters on the health system in order to provide estimated costs for future potential emergencies for national budgetary planning purposes.	18	6.1	(4.3-7.9)	0.9
	**Information and Knowledge Management (1)**				
2	HEDRM Component 5 Information and Knowledge Management: It is important to work in collaboration with academic and research institutions to develop reliable workforce data, such as establishing/strengthening a national and international health workforce observatory.	17	6.6	(5.6-7.6)	0.5
	**Risk Communications (3)**				
2	HEDRM Component 6 Risk Communications: It is important to develop risk communication strategies and build a platform for risk communication useful during public health emergencies for effective coordination with all target groups to support and increase surge capacity.	17	6.7	(5.5-7.9)	0.6
2	HEDRM Component 6 Risk Communications: It is important to include the use of digital technology and telemedicine as an important means to maintain health care service provision during health emergencies.	17	6.2	(4.6-7.8)	0.8
1	HEDRM Component 6 Risk Communications: It is important to include the use of digital information technologies, such as mobile phone applications (mobile apps) and social media in enhancing workforce education or training in Health EDRM as well as public education for disaster risk reduction.	18	6.1	(4.7-7.5)	0.7
	**Health and Related Services (4)**				
2	HEDRM Component 7 Health and Related Services: It is important to include local clinicians and community health workers in disaster preparedness plans and provide trainings to engage and empower primary care workers with disaster response.	17	6.8	(5.8-7.8)	0.5
1	HEDRM Component 7 Health and Related Services: It is important to encourage hospitals to develop and establish their own hospital disaster management plans or the Business Continuity Plan (BCP) in alignment with national-level hospital disaster management guidelines or checklists. Standard operating procedures for all the hospital staff should clarify roles, responsibilities, and action plans of each category of staff (eg, clinical, nonclinical, management) during emergencies and explain when to activate and deactivate each group.	18	6.6	(5.4-7.8)	0.6
1	HEDRM Component 7 Health and Related Services: It is important to review hospital surge capacity regularly and develop systems to identify health workforce personnel available for rapid deployment.	18	6.5	(4.9-8.1)	0.8
2	HEDRM Component 7 Health and Related Services: It is important to develop guidelines or checklists for hospital disaster management plans with standard operating procedures to scale-up service delivery in order to meet increased health needs in hospitals during emergencies. These guidelines and checklists should be consistent with global Health EDRM management workforce frameworks and be customized to specific country contexts.	17	6.4	(5.2-7.6)	0.6
	**Community Capacities for Health EDRM (6)**				
1	HEDRM Component 8 Community Capacities for Health EDRM: In order to increase the community’s coping capacity and resilience during health emergencies, it is important to consider developing community-based workforce development programs with active community involvement from the planning stage.	18	6.6	(5.0-8.2)	0.8
1	HEDRM Component 8 Community Capacities for Health EDRM: It is important to integrate community health workers into disaster risk management structures to promote employment, supervision, support, training, and career development. The roles and responsibility of Community Health Workers should be identified and integrated in health systems and emergency management plans.	18	6.3	(4.9-7.7)	0.7
1	HEDRM Component 8 Community Capacities for Health EDRM: It is important to recognize people in communities, local community leaders, and community health workers as first responders and important members of the health workforce during health emergencies and clearly define their roles and responsibilities in national disaster risk management planning.	18	6.3	(4.7-7.9)	0.8
1	HEDRM Component 8 Community Capacities for Health EDRM: In disaster preparedness phase, it is important to develop a volunteer management policy at national level and establish coordination mechanisms for spontaneous volunteer groups to ensure consistency in the quality-of-service delivery while considering legal and medical protection and training issues.	18	6.2	(4.4-8.0)	0.9
2	HEDRM Component 8 Community Capacities for Health EDRM: It is important to establish volunteer management plans and systems in the disaster preparedness phase to facilitate leadership and gain acceptance in the community. This system should undergo legal review and link up with other disaster management structures.	17	6.2	(4.4-8.0)	0.9
1	HEDRM Component 8 Community Capacities for Health EDRM: In order to strengthen country-led coordination mechanisms to maximize potential contributions from community health workers during health emergencies, it is important to develop strong leadership from the public sector.	18	6.1	(4.7-7.5)	0.7
	**Monitoring and Evaluation (2)**				
1	HEDRM Component 9 Monitoring and Evaluation: It is important to support a national platform to develop and disseminate research evidence and after-action reports to ensure that the research progress is integrated into existing health sector monitoring systems to optimize future responses.	18	6.6	(5.4-7.8)	0.6
1	HEDRM Component 9 Monitoring and Evaluation: It is important to define goals and objectives for health workforce for Health EDRM in a country, in alignment with international frameworks, and develop/establish a system to monitor the goals and objectives.	18	6.4	(5.2-7.6)	0.6

Abbreviations: HEDRM, health emergency and disaster risk management; LMIC, low- and middle-income country.

**Table 4.** Statements Attaining Consensus in the HIC Group

	HIC attained consensus in 34 (out of total 53) statements				
**Round**	**Statement**	**n**	**Mean**	**(95% CI)**	**SD**
	**Policies, Strategies, and Legislation (7)**				
1	HEDRM Component 1 Policies, Strategies, and Legislation: It is important to develop national-level health workforce database system to assess and manage the national-level health workforce, which should be regularly updated, easily accessible, and ready for use during health emergencies.	13	6.2	(4.6-7.8)	0.8
3	HEDRM Component 1 Policies, Strategies, and Legislation: It is important to ensure that workforce strategies give emphasis to strengthening the roles, capacities, and competencies of leaders and managers who are responsible for coordination of health emergency management systems at national, regional, and local levels.	12	6.0	(4.4-7.6)	0.8
3	HEDRM Component 1 Policies, Strategies, and Legislation: It is important to develop Emergency Medical Teams (EMTs) in compliance with the national health system and its legal framework as well as with the WHO EMT standard.	12	6.0	(4.4-7.6)	0.8
3	HEDRM Component 1 Policies, Strategies, and Legislation: It is important to ensure that the health workforce strategy for Health EDRM is aligned with all hazards health EDRM risk management strategies.	12	5.9	(3.9-7.9)	1.0
2	HEDRM Component 1 Policies, Strategies, and Legislation: It is important to develop and implement comprehensive health workforce policies and strategic plans for health emergencies based on good understanding of national labor market dynamics through regular capacity and gap assessments.	12	5.8	(4.4-7.2)	0.7
2	HEDRM Component 1 Policies, Strategies, and Legislation: It is important to ensure that the skills and competencies of public health personnel are up to date to ensure core public health services are maintained throughout a health emergency.	12	5.7	(3.9-7.5)	0.9
2	HEDRM Component 1 Policies, Strategies, and Legislation: It is important to develop workforce strategies at national level to address the full range of capacities in accordance with the WHO Health EDRM framework (details in annex 2) to prevent, prepare, respond, and recover from the range of risks in the country.^15^	12	5.4	(3.8-7.0)	0.8
	**Planning and Coordination (3)**				
2	HEDRM Component 2 Planning and Coordination: It is important to develop strategies to reduce stressors in order to support the mental health and well-being of the health workforce during emergencies, such as securing a sustainable supply of workers, providing disaster risk management training, and establishing clear response guidelines and safety protocols.	12	6.2	(4.4-8.0)	0.9
2	HEDRM Component 2 Planning and Coordination: It is important to develop strategies for achieving optimal task-shifting, involving national-level planning, investment, training, quality assurance, and coordination with external supporting agencies.	12	5.8	(4.0-7.6)	0.9
2	HEDRM Component 2 Planning and Coordination: It is important to conduct regular health workforce capacity and gap assessments to understand national labor market dynamics for achieving optimal workforces with respect to quantity, quality, and availability during health emergencies.	12	5.7	(4.3-7.1)	0.7
	**Human Resources (7)**				
3	HEDRM Component 3 Human Resources: It is important to ensure that the content of national-level education courses and training programs for Health EDRM are based on an all-hazards approach to protect the physical, mental, and social well-being of the affected people. However, the content should also include specific health risks and anticipated impact from high-likelihood events in a country for their frontline workers and their first response organizations.	12	6.0	(4.0-8.0)	1.0
3	HEDRM Component 3 Human Resources: It is important to implement national-level disaster training courses with multidisciplinary approach to bring different knowledge and expertise together during health emergencies in order to maximize the use of available resources and systems to save people’s lives.	12	5.9	(3.9-7.9)	1.0
2	HEDRM Component 3 Human Resources: It is important to work in collaboration with existing national/international professional organizations to develop national/regional/international training programs for Health EDRM.	12	5.7	(3.9-7.5)	0.9
3	HEDRM Component 3 Human Resources: It is important to identify core competencies for the key emergency management posts in the health ministry and should ensure their alignment with country’s national disaster risk management plan.	12	5.5	(3.5-7.5)	1.0
3	HEDRM Component 3 Human Resources: It is important to establish a quality assurance mechanism for Health EDRM training programs or education courses, by monitoring and evaluating course achievements and by revising the course contents and modes of delivery according to participants’ needs.	12	5.2	(3.8-6.6)	0.7
3	HEDRM Component 3 Human Resources: It is important to develop competency-based disaster education courses and training programs for all workforce required during health emergencies, including nurses, paramedics, doctors, hospital managers, and administrative workers.	12	5.2	(3.6-6.8)	0.8
3	HEDRM Component 3 Human Resources: It is important to develop national-level education courses and training programs for Health EDRM in compliance with internationally recognized core competency standards adapted according to national health system practice and priorities.	12	5.1	(3.5-6.7)	0.8
	**Financial Resources (3)**				
3	HEDRM Component 4 Financial Resources: It is important to allocate sufficient financial resources and invest in the health workforce in order to meet current and future needs during health emergencies for a country.	12	6.2	(4.2-8.2)	1.0
2	HEDRM Component 4 Financial Resources: It is important to allocate sufficient contingency funding for increased workforce costs in all phases of disasters.	12	5.8	(4.0-7.6)	0.9
3	HEDRM Component 4 Financial Resources: It is important to establish mechanisms and platforms to document and share the economic impacts of previous disasters on the health system in order to provide estimated costs for future potential emergencies for national budgetary planning purposes.	12	5.2	(3.4-7.0)	0.9
	**Information and Knowledge Management (2)**				
2	HEDRM Component 5 Information and Knowledge Management: It is important to work in collaboration with academic and research institutions to develop reliable workforce data, such as establishing/strengthening a national and international health workforce observatory.	12	6.1	(4.5-7.7)	0.8
3	HEDRM Component 5 Information and Knowledge Management: It is important to establish a database for EMTs including reliable information about skills, abilities, and availabilities of staff trained in emergency management. These data should be maintained by national government or designated organizations and be readily available during emergencies as a part of Emergency Response coordination structure.	12	5.5	(3.5-7.5)	1.0
	**Risk Communications (3)**				
2	HEDRM Component 6 Risk Communications: It is important to develop risk communication strategies and build a platform for risk communication useful during public health emergencies for effective coordination with all target groups to support and increase surge capacity.	12	6.2	(4.4-8.0)	0.9
3	HEDRM Component 6 Risk Communications: It is important to include the use of digital technology and telemedicine as an important means to maintain health care service provision during health emergencies.	12	5.4	(3.4-7.4)	1.0
3	HEDRM Component 6 Risk Communications: It is important to include the use of digital information technologies, such as mobile phone applications (mobile apps) and social media in enhancing workforce education or training in Health EDRM as well as public education for disaster risk reduction.	12	5.4	(3.4-7.4)	1.0
	**Health and Related Services (3)**				
3	HEDRM Component 7 Health and Related Services: It is important to include local clinicians and community health workers in disaster preparedness plans and provide trainings to engage and empower primary care workers with disaster response.	12	6.2	(5.0-7.4)	0.6
3	HEDRM Component 7 Health and Related Services: It is important to develop guidelines or checklists for hospital disaster management plans with standard operating procedures to scale up service delivery, in order to meet increased health needs in hospitals during emergencies. These guidelines and checklists should be consistent with global Health EDRM management workforce frameworks and be customized to specific country contexts.	12	5.8	(4.2-7.4)	0.8
2	HEDRM Component 7 Health and Related Services: It is important to review hospital surge capacity regularly and develop systems to identify health workforce personnel available for rapid deployment.	12	5.5	(3.7-7.3)	0.9
	**Community Capacities for Health EDRM (4)**				
3	HEDRM Component 8 Community Capacities for Health EDRM: It is important to integrate community health workers into disaster risk management structures to promote employment, supervision, support, training, and career development. The roles and responsibility of Community Health Workers should be identified and integrated in health systems and emergency management plans.	12	5.8	(3.8-7.8)	1.0
2	HEDRM Component 8 Community Capacities for Health EDRM: It is important to recognize people in communities, local community leaders, and community health workers as first responders and important members of the health workforce during health emergencies and clearly define their roles and responsibilities in national disaster risk management planning.	12	5.7	(3.9-7.5)	0.9
2	HEDRM Component 8 Community Capacities for Health EDRM: In disaster preparedness phase, it is important to develop a volunteer management policy at national level and establish coordination mechanisms for spontaneous volunteer groups to ensure consistency in the quality-of-service delivery while considering legal and medical protection and training issues.	12	5.2	(3.4-7.0)	0.9
3	HEDRM Component 8 Community Capacities for Health EDRM: In order to strengthen country-led coordination mechanisms to maximize potential contributions from community health workers during health emergencies, it is important to develop strong leadership from the public sector.	12	5.2	(3.2-7.2)	1.0
	**Monitoring and Evaluation (2)**				
2	HEDRM Component 9 Monitoring and Evaluation: It is important to support a national platform to develop and disseminate research evidence and after-action reports to ensure that the research progress is integrated into existing health sector monitoring systems to optimize future responses.	12	5.8	(4.0-7.6)	0.9
3	HEDRM Component 9 Monitoring and Evaluation: It is important to define goals and objectives for health workforce for Health EDRM in a country, in alignment with international frameworks, and develop/establish a system to monitor the goals and objectives.	12	5.3	(3.5-7.1)	0.9

Abbreviations: EMT, Emergency Medical Team; HEDRM, health emergency and disaster risk management; HIC, high-income country; WHO, World Health Organization.

## Discussion

This expert consensus study identified strategic recommendations for strengthening workforce capacities for disasters and health emergencies in both HIC and LMIC settings. Consensus was achieved for 34 recommendation statements in the HIC group and 44 statements in the LMIC group. These strategic recommendations can be used to guide future Health EDRM workforce capacity building before, during, and after disasters. The high means on the one-to-seven linear rating scale (5.9 to 6.8 for the LMIC group and 5.1 to 6.2 for the HIC group) reflect the high level of importance accorded to all statements attaining consensus. Furthermore, there were a number of statements that did not meet the cut-off point for consensus but received a high mean score for importance. They should not be regarded as being unimportant or not needed, rather they did not reach the level of consensus as other statements.

### Investing in the Health EDRM Workforce

The results show that participants felt strongly that human resource capacities and management systems are crucial to many Health EDRM functions. A risk management approach to Health EDRM recognizes that the entire health workforce has roles to play to reduce the risk and impact of emergencies and disasters. The importance to allocate sufficient financial resources and investment in the health workforce was recognized by both LMIC and HIC groups.

The WHO defined the health workforce as “all people engaged in actions whose primary intent is to enhance health.”^42^ This definition includes all health professionals who are essential for maintaining functional health systems, as well as workforce groups not traditionally regarded to be in the health domain, such as rescue personnel, police, and community health workers.^43^ To effectively manage and mobilize all available human resources with different skill sets, experiences, and knowledge, a good understanding of the wide-ranging composition of the Health EDRM workforce is necessary.

Due to the diverse composition of the Health EDRM workforce, the need for well-established models for administration and governance of systems and programs at the country level and the co-dependencies for fulfilling functions, there are intrinsic difficulties in providing a robust taxonomy for the Health EDRM workforce; for example, based on workforce groups. Several key recommendations in both the LMIC and the HIC groups emphasized the importance of health workforce capacity and gap assessments of the national labor market, developing national-level health workforce database systems, and collaborating with academic and research institutions to establish national and international health workforce observatories.

### Adapting to the Local Context – Results from the LMIC and HIC Groups

Consensus was attained for 44 recommendations in the LMIC group (mean of mean scores 6.4; range 5.9 to 6.8), suggesting that most of statements were regarded as important in LMIC settings. There were fewer statements that attained consensus in the HIC group (34 statements), and those that did achieve consensus did so with a lower score (mean of mean scores 5.7; range 5.1 to 6.2).

There were also differences in the number of final recommendations by Health EDRM component. A higher number of statements attained consensus and high mean score in the “Policies, Strategies, and Legislation” and “Information and Knowledge Management” component in the HIC group compared with the LMIC group. On the other hand, in the LMIC group, there were more statements that achieved consensus and high mean score in the components of “Planning and Coordination,” “Human Resources,” “Community Capacities for Health EDRM,” and to a lesser extent, in “Health and Related Services.” These differences could be influenced by factors such as the level of national disaster risk management capacities, available financial resources, or having established professional bodies and health systems. Many HICs may already have established national disaster risk management strategies and plans, so they may have different needs when it comes to the recommendation statements included in the survey.

The fewer number of statements attaining consensus in the HIC group may have been due to larger differences in Health EDRM in HICs. It may suggest that priorities in LMIC are more systemic, with fundamental challenges stemming from their health systems compared to HICs. Workforce challenges for the Health EDRM workforce are similar to those for health systems in general (eg, availability, accessibility, employment, recruitment, remuneration, retention, and occupational health and safety). In fact, these challenges are likely to be amplified during emergencies and disasters, as the COVID-19 pandemic has highlighted.^44^ The integration of “Health EDRM” into overall health workforce strategies is needed in many countries, but may be more needed in LMIC, judging by the statements reaching consensus.

It is interesting to note that most of the EMT statements were de-prioritized by the LMIC group but reached consensus in the HIC group. Some panelists reflected that EMTs, while being very relevant to readiness and medical response to disasters, are just a portion of the many other “teams” and “health disciplines” that form Health EDRM. This may be related to the fact that EMT initiative built on the earlier work of “foreign medical teams” that focused on support provided by HIC countries, and the concept of EMTs may not yet have been fully translated across the LMIC settings.

### Integrating with a Health Systems Approach

Given current and emerging threats (such as disease outbreaks, climate-related hazards, violence, and conflict) as well as vulnerabilities (such as poverty, inequities within and between countries, lack of access to primary health care, and emergency department over-crowding), narrowing the selection of strategic recommendations was challenging. In Phase 1 of this study, the results of a literature review that evaluated strategic workforce management and drew capacity building recommendations^23^ was modified to adapt to the Health EDRM context following the “all-hazard” and “whole-of-society” approach.^17,34^ The need for the integration of these emergency preparedness efforts with a health systems approach was also highlighted in the results of this study.

Notably, the importance of risk communication strategies and establishing risk communication platforms was reflected in one of the statements attained consensus and high mean score both for the LMIC and the HIC group: “*To develop risk communication strategies and build a risk communication platform for effective coordination with all target groups during public health emergencies and support and increase surge capacity.*” The importance of workforce development in this area in the recent COVID-19 pandemic was also observed.^45^

The recommendation to “*Ensure that the content of national-level education courses and training programs for Health EDRM are based on an all-hazards approach to protect the physical, mental, and social well-being of the affected people*” was also among the statements attaining consensus and high mean score for both LMIC and HIC groups. While there remained a lack of universally accepted set of core competencies for the disaster health care providers,^46,47^ efforts are underway to provide common learning strategies for health emergencies programs such as in the WHO.^48^

Lastly, the importance of engaging and equipping the primary health care workforce and community health workers is reflected in the recommendation to “*Include local clinicians and community health workers in disaster preparedness plans and provide trainings to engage and empower primary care workers with disaster response*” attaining consensus in both LMIC and HIC group. This effort is synonymous with the WHO Thirteenth General Program of Work (GPW 13) and its target of achieving universal health coverage.^49^ A “health systems” approach is required to ensure that the fundamental elements of Health EDRM in health workforce development are addressed.

## Limitations

This study had a few limitations. There are no universally agreed upon definitions of consensus for Delphi studies, and the analysis of Likert-type scales remains controversial. The one-to-seven linear scale was used in this study, which is different from Likert scale and anchored only on the extremes. The design of the one-to-seven linear scale considers that the distance between each of the numbers is equal and allow the use of parametric statistical tests.^40^ The definition of consensus and importance using SD and mean was based on this reasoning.

There are also no set criteria on how panelists should be selected. As “experts” were not selected randomly, generalization of the results may be limited.^50^ As the panelists in Phase 1 and Phase 2 were mostly from academic institutions, there is a risk that their ratings might have been skewed toward more academic research directions. It is important to note that consensus may have been influenced by the way in which panelists responded to the survey (depending on their levels, roles, and functions), as the understanding of Health EDRM varies across respondent groups. For example, there may have been variations in the way people at the sub-national, national, and supra-national levels responded. Panelists could also be swayed by the most recent disaster (eg, the immediacy of the COVID-19 experience may be a source of bias towards disasters associated with infectious diseases).

Required strategies in each country can vary depending on the national context (eg, level of capacities for disaster preparedness and response, political structure, available resources, disaster risks, or health systems). Each country’s strategy to develop and strengthen the health workforce for Health EDRM will depend on the national context in terms of disaster risks, available resources, experience, or governance systems. It is important to note that even though there were recommendations that did not attain consensus, these may still be important strategies for a particular country’s profile. It is also important to recall that consensus and the use of standard deviation results in a set of priorities, but that is not to say that those statements that did not reach this level of consensus are “unimportant” or “not needed.”

## Conclusion

The expert panel provided a comprehensive list of important and actionable strategic recommendations on workforce development for Health EDRM. Health EDRM workforce strategies should emphasize strengthening the roles, capacities, and competencies of leaders and managers who are responsible for leading, developing, and coordinating systems and programs for Health EDRM.

## Supplementary material

For supplementary material accompanying this paper visit https://doi.org/10.1017/S1049023X22001467. click here to view supplementary material
