# Supplementary material for: Health Emergency and Disaster Risk Management Workforce Development Strategies: Delphi Consensus Study
Source: Prehosp Disaster Med. 2022 Nov 3;37(6):735–48. doi: 10.1017/S1049023X22001467 (PMC9726469; doi:10.1017/S1049023X22001467)
Supplement: Supplementary file 1 [file S1049023X22001467sup001.docx]

**Supplementary Table 1: Overview of the Expert Consensus Study**

| **Expert Consensus Study** | | | |
| --- | --- | --- | --- |
| Gain consensus among global experts on developing  national-level workforce strategies for health emergencies | | | |
|  | **PHASE ONE** | **PHASE TWO** | |
| **Aims** | Review and finalise recommendation statements for a Delphi survey in PHASE TWO | Expert consensus study using a modified Delphi approach | |
| **Panellists** | 14 Research Project Members | 31 global experts in Health EDRM | |
|  |  | LMIC Group 18 | HIC Group 13 |
| **Methods** | Reviewing 51 preselected statements via a digital survey | 3 rounds of surveys | 3 rounds of surveys |
| **Outputs** | 46 recommendation statements | - - 44 consensus attained statements   - 8 consensus non-attained statements | - - 34 consensus attained statements   - 19 consensus non-attained statements |

**Supplementary Table 2: Summary of Three Rounds of the Delphi Surveys**

|  | **1st Survey Summary** | | | **2st Survey Summary** | | | **3rd Survey Summary** | | | **Total** | | |
| --- | --- | --- | --- | --- | --- | --- | --- | --- | --- | --- | --- | --- |
| Dates | From 02/08/2021 to 23/08/2021 | | | From 24/08/2021 to 13/09/2021 | | | From 13/09/2021 to 03/10/2021 | | |  | | |
|  |  | **HIC Group** | **LMIC Group** |  | **HIC Group** | **LMIC Group** |  | **HIC Group** | **LMIC Group** |  |  |  |
| **Experts** | Invited | 17 | 24 | Invited | 17 | 24 | Invited | 17 | 24 |  |  |  |
|  | Responded | 13 | 18 | Responded | 12 | 17 | Responded | 12 | 15 |  |  |  |
|  | Not responded | 4 | 6 | Not responded | 5 | 7 | Not responded | 5 | 9 |  |  |  |
| **Statements** | Included | 46 | 46 | Included | 51 | 22 | Included | 38 | 11 |  | **HIC Group** | **LMIC Group** |
|  | Attained | 1 | 28 | Attained | 14 | 13 | Attained | 19 | 3 | Attained | 34 | 44 |
|  | Not Attained | 45 | 18 | Not Attained | 37 | 9 | Not Attained | 19 | 8 | Not Attained | 19 | 8 |
|  | New statements | 6 | 4 | New statements | 1 | 2 |  |  |  | Total | 53 statements  (46 initial + 7) | 52 statements  (46 initial + 6) |
|  | For the 2nd round | 51 | 22 | For the 3rd round | 38 | 11 |  |  |  |  |  |  |

**Supplementary Table 3: Statements not attained consensus in the LMIC group**

|  | **LMIC Not Attained (8)** |  |  |  |  |  |
| --- | --- | --- | --- | --- | --- | --- |
| **Round** | **Statement** | **n** | **Mean** | **95% CI** | | **SD** |
| 3 | HEDRM Component 1 Policies, Strategies and Legislation: It is important to ensure that all workforce for emergency and disaster management should be skilled with a rights-based risk management approach. | 15 | 6.3 | 4.1- | 8.5 | 1.1 |
| 3 | HEDRM Component 1 Policies, Strategies and Legislation: It is important to provide guidance on team composition, skills required, deployment mechanisms, logistics for national and local Emergency Medical Teams, in compliance with the WHO EMT standard but adopted to the local needs based on the types of local specific disasters, potential health risks, and geographical characteristics for all types and phases of disasters. | 15 | 5.9 | 3.1- | 8.7 | 1.4 |
| 3 | HEDRM Component 1 Policies, Strategies and Legislation: It is important to develop Emergency Medical Teams (EMTs) in compliance with the national health system and its legal framework as well as with the WHO EMT standard. | 15 | 5.9 | 2.9- | 8.9 | 1.5 |
| 3 | HEDRM Component 2 Planning and Coordination: It is important to establish tested chains of command and control structures at national level in deploying and organising Emergency Medical Teams and, coordinating with international actors. | 15 | 6.2 | 3.8- | 8.6 | 1.2 |
| 3 | HEDRM Component 2 Planning and Coordination: It is important to ensure that coordination mechanisms between Emergency Medical Teams and general medical service are in place for continuity of care/smooth transfer from hyperacute and acute phase emergency medical care to subacute or chronic phase general medical care. | 15 | 6.2 | 3.2- | 9.2 | 1.5 |
| 3 | HEDRM Component 2 Planning and Coordination: It is important to develop emergency response coordination structures and mechanisms at national level for deploying national Emergency Medical Teams in collaboration with relevant professional associations in a country, e.g. doctors and nurses professional associations. | 15 | 5.9 | 3.1- | 8.7 | 1.4 |
| 3 | HEDRM Component 3 Human Resources: It is important to ensure that leadership programs at local level are developed and implemented for effective health workforce in disaster risk management. | 15 | 6.3 | 3.3- | 9.3 | 1.5 |
| 3 | HEDRM Component 5 Information and Knowledge Management: It is important to establish a database for Emergency Medical Teams including reliable information about skills, abilities, and availabilities of staff trained in emergency management. These data should be maintained by national government or designated organisations and be readily available during emergencies as a part of Emergency Response coordination structure. | 15 | 6.3 | 3.3- | 9.3 | 1.5 |

**Supplementary Table 4: Statements not attained consensus in the HIC group**

|  | **HIC Not Attained (19)** | |  |  |  |  |  |
| --- | --- | --- | --- | --- | --- | --- | --- |
| **Round** | | **Statement** | **n** | **Mean** | **95% CI** | | **SD** |
| 3 | HEDRM Component 1 Policies, Strategies and Legislation: It is important to provide guidance on team composition, skills required, deployment mechanisms, logistics for National-level and local Emergency Medical Teams, in compliance with the WHO EMT standard but adopted to the local needs based on the types of local specific disasters, potential health risks, and geographical characteristics for all types and phases of disasters. | | 12 | 5.4 | 3.0- | 7.8 | 1.2 |
| 3 | HEDRM Component 1 Policies, Strategies and Legislation: It is important to outline comprehensive workforce structures for health emergencies in the national disaster risk management policy and strategy. | | 12 | 5.3 | 2.9- | 7.7 | 1.2 |
| 3 | HEDRM Component 2 Planning/Coordination: It is important to ensure that coordination mechanisms between Emergency Medical Teams and general medical service are in place for continuity of care/smooth transfer from hyperacute and acute phase emergency medical care to subacute or chronic phase general medical care. | | 12 | 6.2 | 3.6- | 8.8 | 1.3 |
| 3 | HEDRM Component 2 Planning and Coordination: It is important to develop context-specific well-rehearsed Mass casualty incidents (MCIs) protocols, guidelines and management systems to facilitate an immediate systematic response to MCIs in order to continuously operate rapid and timely deployment of trained personnel to needed areas in hospitals and prehospital (on-site) settings. | | 12 | 6.0 | 3.8- | 8.2 | 1.1 |
| 3 | HEDRM Component 2 Planning/Coordination: It is important to establish tested chains of command and control structures at national level in deploying and organising Emergency Medical Teams and, coordinating with international actors. | | 12 | 5.8 | 2.4- | 9.2 | 1.7 |
| 3 | HEDRM Component 2 Planning/Coordination: It is important to develop emergency response coordination structures and mechanisms at national level for deploying national Emergency Medical Teams in collaboration with relevant professional associations in a country, e.g. doctors and nurses professional associations. | | 12 | 5.6 | 3.0- | 8.2 | 1.3 |
| 3 | HEDRM Component 2 Planning and Coordination: It is important to establish health professional license verification mechanism for foreign health professional volunteers in disaster preparedness phase. | | 12 | 5.3 | 2.9- | 7.7 | 1.2 |
| 3 | HEDRM Component 2 Planning and Coordination: It is important to encourage the development of systems or mechanisms to monitor stress levels and stressors and to provide mental health and psychosocial support to the healthcare workforce during health emergencies. | | 12 | 5.3 | 1.9- | 8.7 | 1.7 |
| 3 | HEDRM Component 2 Planning/Coordination: It is important to develop emergency response coordination structures and mechanisms with international humanitarian actors to maximise workforce capacity in response planning, and service delivery including deployment and coordination mechanism in a way to support building back better of affected local health system. | | 12 | 4.8 | 1.8- | 7.8 | 1.5 |
| 3 | HEDRM Component 3 Human Resources: It is important to consider a multidisciplinary approach with professionals from various fields for training national / international Emergency Medical Teams to perform effective operations in emergency settings. | | 12 | 5.9 | 3.3- | 8.5 | 1.3 |
| 3 | HEDRM Component 3 Human Resources: In order to increase the willingness and remove barriers of workers to respond in disasters, it is important to consider developing a system to provide family care support and other modifiable factors. | | 12 | 5.8 | 3.6- | 8.0 | 1.1 |
| 3 | HEDRM Component 3 Human Resources: It is important to prioritise on providing timely payment of salaries and ensuring occupational safety in order to increase retention and motivation. | | 12 | 5.7 | 2.1- | 9.3 | 1.8 |
| 3 | HEDRM Component 3 Human Resources: It is important to consider a blended learning approach for national level education courses and training programmes for Health EDRM. The combination of different teaching methods such as traditional instructor-led teaching and technology-enhanced learning, table-top exercises, and computerised and real-world simulations is useful to stimulate different sets of skills. | | 12 | 5.2 | 2.8- | 7.6 | 1.2 |
| 3 | HEDRM Component 3 Human Resources: It is important to develop a formal mechanism at national level, e.g. needs assessment or skill-gap assessment, to assess training needs regularly. Training institutes should periodically evaluate and update these needs-oriented, competency-based curricula and training activities. | | 12 | 5.1 | 2.7- | 7.5 | 1.2 |
| 3 | HEDRM Component 3 Human Resources: It is important to develop education courses and training programmes for Health EDRM. These courses and programmes require clear aims and learning objectives in alignment with country's needs and should be based on nationally/internationally/ recognised organisation standards. | | 12 | 5.1 | 2.1- | 8.1 | 1.5 |
| 3 | HEDRM Component 3 Human Resources: It is important to identify adapted definitions of workforce in public health emergencies for their country and clearly define their roles and responsibilities. The required characteristics and scope of those involved may differ depending on country's contexts according to health systems, legal and ethical framework, disaster risk management planning and coordination mechanisms, human resource management, financing, and governance mechanisms. | | 12 | 4.9 | 2.3- | 7.5 | 1.3 |
| 3 | HEDRM Component 7 Health and Related Services: It is important to encourage hospitals to develop and establish their own hospital disaster management plans or the Business Continuity Plan (BCP) in alignment with national level hospital disaster management guidelines or checklists. Standard operating procedures for all the hospital staff should clarify roles, responsibilities and action plans of each category of staff (e.g. clinical, nonclinical, management) during emergencies and explain when to activate and deactivate each group. | | 12 | 6.0 | 3.8- | 8.2 | 1.1 |
| 3 | HEDRM Component 8 Community Capacities for Health EDRM: In order to increase the community's coping capacity and resilience during health emergencies, it is important to consider developing community-based workforce development programmes with active community involvement from the planning stage. | | 12 | 5.5 | 3.3- | 7.7 | 1.1 |
| 3 | HEDRM Component 8 Community Capacities for Health EDRM: It is important to establish volunteer management plans and systems in the disaster preparedness phase, to facilitate leadership and gain acceptance in the community. This system should undergo legal review, and link up with other disaster management structures. | | 12 | 4.8 | 1.8- | 7.8 | 1.5 |
